# Association of the Charlson Comorbidity Index with Renal Outcome and All-Cause Mortality in Antineutrophil Cytoplasmatic Antibody-Associated Vasculitis

**DOI:** 10.1097/MD.0000000000000152

**Published:** 2014-11-28

**Authors:** Shachaf Ofer-Shiber, Yair Molad

**Affiliations:** From the Department of Internal Medicine (SO-S); Rheumatology Unit, Beilinson Hospital, Rabin Medical Center (YM); and Sackler Faculty of Medicine, Tel Aviv University (SO-S, YM), Tel Aviv, Israel.

## Abstract

The aim of this study is to determine the effect of comorbidity assessed by the Charlson comorbidity index (CCI) at the time of diagnosis on the outcome of antineutrophil cytoplasmatic antibody (ANCA) associated vasculitis (AAV).

This is a longitudinal observational study of 30 consecutive patients with AAV who were diagnosed and followed from January 1996 to December 2011. The degree of comorbidity at diagnosis and last visit was scored according to the age-adjusted Charlson comorbidity index (CCI (a)). The post hoc analysis of increment in CCI during the study period and its predictive value for patient and renal survival were analyzed.

Thirty patients with AAV were included in this study. A higher CCI (a) at diagnosis was positively correlated with higher activity score of AAV (*P* = 0.016), a CCI (a) >5, and with an increased risk for mortality (odds ratio 12; confidence interval 1.8–79.68, *P* = 0.014). The mean increment (Δ) of CCI (a) during the study period was 1.26 ± 2.03 (6–5). Correlation was found between lower Δ CCI (a) and chronic kidney disease (*P* = 0.036) and mortality (*P* = 0.002).

Comorbidity at the time of diagnosis of AAV is associated with reduced patient and renal survival. We suggest including the CCI score in the assessment of patients with AAV at diagnosis and at disease relapse.

## INTRODUCTION

Comorbidities are very common among rheumatic patients.^1–3^ There is an increased incidence of comorbidities in rheumatic patients due to the inflammatory process as well as to the adverse effects of treatment.

There are many tools in medicine to quantify comorbidity and prognosis. The most famous is the Charlson comorbidity index (CCI), which is considered the gold standard for the assessment of comorbidity risk in clinical research.^4^

The CCI is calculated by summing the weights for each condition in the medical history. In 1994, a modification of the CCI, which considers the effect of aging on mortality was published (age-adjusted Charlson comorbidity index [CCI (a)])^5^ that adds an extra point for each decade of age above 50 years to the original CCI.

Most studies with respect to comorbidities in patients with rheumatic diseases were conducted on rheumatoid arthritis patients.^6,7^ In lupus, a high CCI was associated with decreased survival independent of age, lupus disease activity, and damage.^8^

Antineutrophil cytoplasmatic antibody (ANCA) associated vasculitis (AAV) is a heterogeneous group of diseases corresponding to necrotizing inflammation of small vessels mostly affecting the respiratory system, kidneys, nervous system, and skin. AAV includes granulomatosis with polyangiitis (GPA), eosinophilic GPA (EGPA) and microscopic polyangiitis, as well as ANCA-associated isolated organ disease.^9^ CCI has not been studied in patients with vasculitis.

In the present study, we sought to determine the effect of comorbidity assessed by the CCI on the outcome of patients with AAV.

## METHODS

This is a longitudinal observational study of 30 consecutive patients with AAV, who were diagnosed with AAV at our hospital from January 1996 to December 2011. All patients met the criteria of Chapel-Hill Consensus Conference definition for AAV.^10^ All patients were routinely followed at our Vasculitis Clinic and were treated according to the attending physician's discretion. Patients’ hospital charts and electronic data were retrospectively and systematically analyzed for the demographic (age, gender, origin), clinical features at presentation, treatment, and outcome. Specifically, we analyzed the following laboratory data that have been obtained at the time of AAV diagnosis and during follow-up clinic visits: erythrocyte sedimentation rate, c-reactive protein (CRP), antiproteinase 3 (PR3) and antimyeloperoxidase (MPO) antibody, white blood count, hemoglobin, serum creatinine, urinalysis for urine protein, red blood count and casts, as well as 24-hour urine protein excretion. ANCA tests were performed at the hospital immunology laboratory by an antigen-specific enzyme-linked immunosorbent assay at the time of presentation and during follow-up visits. Estimated glomerular filtration rate (eGFR) was calculated for each patient for the time of diagnosis and for the last visit of the study period using the modification of diet in renal disease equation. Chronic kidney disease (CKD) was defined as an eGFR <60 mL/min/1.73 m^2^. For each patient, we determined the five factor score (FFS) that had been developed by the French Vasculitis Study Group to predict the risk of death of patients with systemic vasculitis.^11^ The FFS is a 5-point score that includes reduced renal function (creatinine >1.58 mg/dL); proteinuria (>1 g/24 h); gastrointestinal hemorrhage, infarction, or pancreatitis; involvement of the central nervous system; or cardiomyopathy. Disease activity at the time of diagnosis and last visit was scored according to the Birmingham Vasculitis Activity Score version 3 (BVAS v.3) that includes a clinical checklist of relevant symptoms, signs, and features of active disease.^12^

We retrospectively quantified comorbidity according to the CCI level for the first (at diagnosis) and last encounter during the study period. We used a modified CCI that excludes connective tissue disease and renal failure because these parameters are AAV associated. We also retrospectively scored a modification of the CCI, which considers the effect of aging on mortality CCI (a) uses the original CCI and 1 extra point for each decade of age above st and first visits (ΔCCI).

### Statistical Analysis

Descriptive statistics were used to illustrate 50 years.^13^ For this study, we have calculated the difference of CCI between the la.

The baseline characteristics of the study population. Chi-square analysis and odds ratio and 95% confidence interval (CI) were calculated to test our hypothesis that CCI in the first visit predicts worse. We also use the Kaplan–Meier survival analysis to examine ΔCCI and mortality (Table 1).

**TABLE 1:**
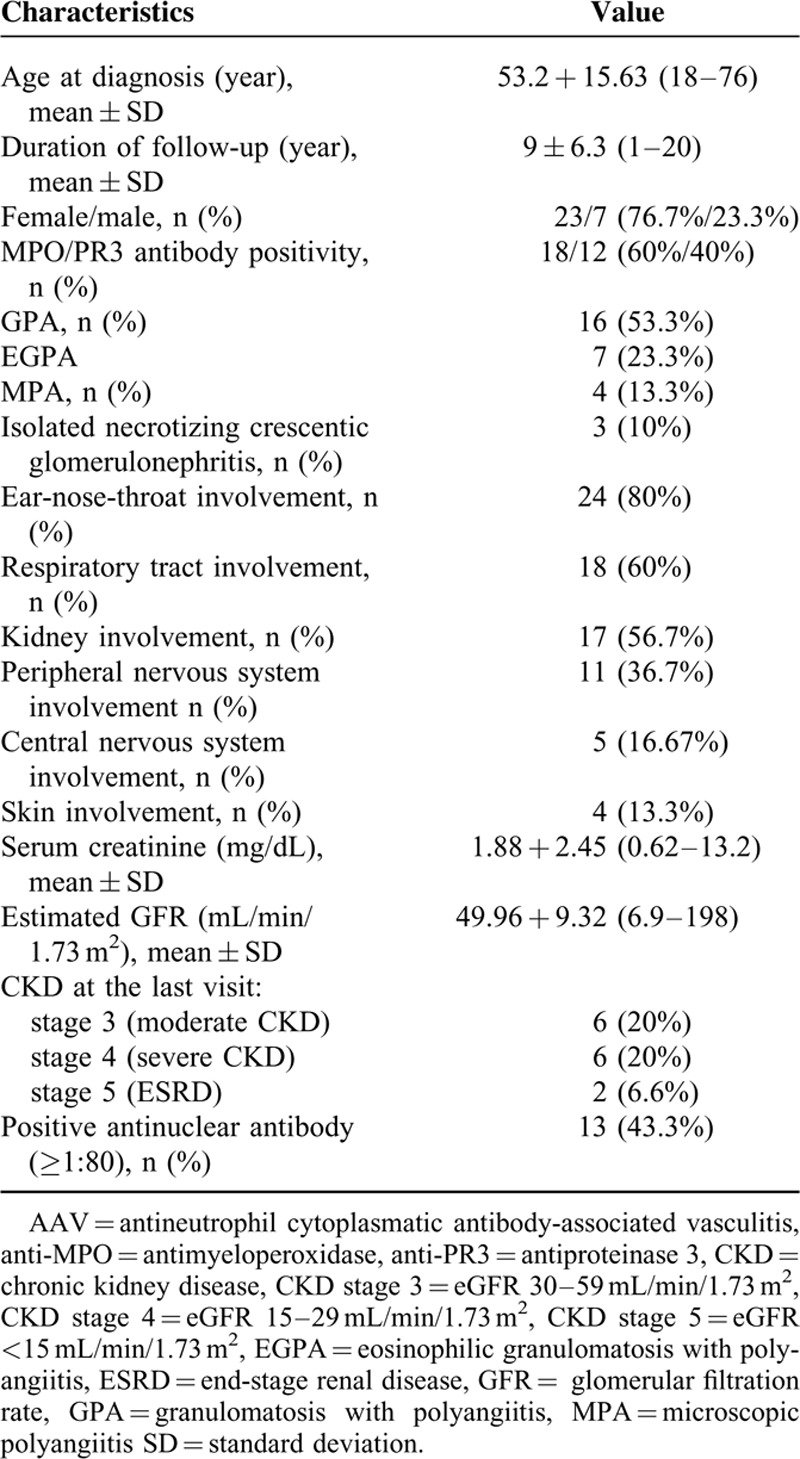
Background, Clinical, and Laboratory Features of 30 Patients With AAV

## RESULTS

### Demographic and Clinical Data at the Time of Disease Presentation

Thirty patients who had been diagnosed with AAC based on the Chapel-Hill criteria^10^ were included in this study. Their mean age at diagnosis was 53.2 ± 15.6 years (range: 18–76 years), 23 (76.7%) women and all of our patients were Jewish (of whom 57.6% Ashkenazi). The mean follow-up period was 9 ± 6.3 years (range: 1–20 year). Sixteen (53.3%) of our cohort had disease presentation compatible with the diagnosis of GPA, 7 (23.3%) with EGPA, 4 (13.3%) with MPA, and 3 (10%) had isolated-organ AAV. Mean serum creatinine at presentation was 1.9 ± 2.45 mg/dL (range: 0.6–13.2 mg/dL) and eGFR 95.9 ± 9.3 mL/min/1.73 m^2^. Eighteen patients (60%) were tested positive for anti-MPO antibody with a mean value of 55.6 ± 85.6 and 12 patients (40%) for anti-PR3 antibody with a mean value of 20.9 ± 30.1 at presentation. The main comorbidities that occurred during the study period were hypertension (12 patients, 40%), diabetes mellitus (7 patients, 23.3%), stroke/transient ischemic attack (2 patients, 6.7%), and coronary heart disease (1 patient, 3.3%). Five patients (16.7%) accrued a malignant disease during the study period, mostly solid tumors (4 patients, 13.3%), and 1 (3.3%) patient with a hematologic malignancy. Five (16.67%) patients died during the follow-up: 1 from malignancy and 4 from infection (Table 2).

**TABLE 2:**
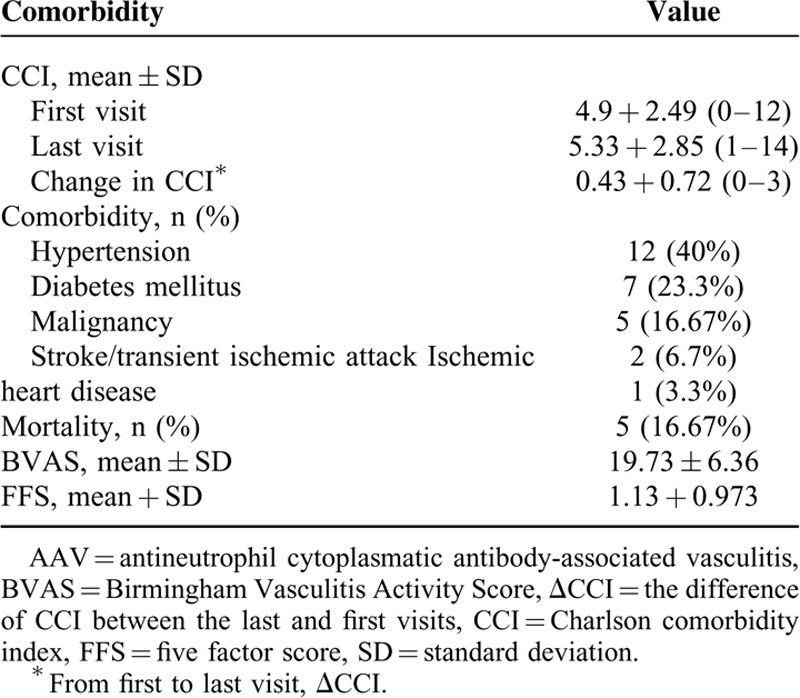
Comorbidity in 30 Patients With AAV

Mean FFS and BVAS at the time of diagnosis was 1.13 ± 0.973 (range: 0–3) and 19.73 ± 6.36 (range: 9–32), respectively.

All of our patients were initially treated with high-dose of oral prednisone with an immunosuppressive treatment (cyclophosphamide with or without maintenance with azathioprine or methotrexate), and trimethoprim-sulfamethoxazole treatment was given to 13 patients (43.3%).

### Association Between CCI At Presentation and Clinical Parameters and Survival

The mean CCI (a) at diagnosis and at the last study encounter was 4.9 ± 2.49 (range: 1–12) and 5.33 ± 2.85 (range: 1–14), respectively. Correlation was found between higher CCI (a) and active disease (*P* = 0.021) at diagnosis and higher CCI (a) at the last study encounter (*P* = 0.001). Disease activity assessed by BVAS was positively correlated with a higher CCI (a) at diagnosis (*P* = 0.02), but not with the FFS. A CCI (a) at diagnosis ≥5 was significantly associated with a greater risk of death (odds ratio 12; CI 1.8–79.68, *P* = 0.014).

### Association Between Increment of CCI at Diagnosis and Last Encounter (ΔCCI (a)) and Clinical Parameters and Patient and Renal Survival

The mean ΔCCI (a) was 1.26 ± 2.03 (range: 6–5). The accrual of CKD defined as eGFR ≤60 mL/min/2.73 m^2^ and mortality were inversely correlated with ΔCCI (a): *P* = 0.036 and *P* = 0.002, respectively, which reflects the grave impact of a higher CCI (a)) at diagnosis on the outcome on renal and patient survival. A ΔCCI (a) of >1 was significantly associated with reduced patients survival 6.9 (CI 1.0063–47.4839, *P* = 0.02).

## DISCUSSION

Patients with vasculitis are at an increased risk of accrual of comorbid conditions either due to disease-associated organ damage and/or due to drugs adverse effects.

The introduction of treatment with cyclophosphamide and corticosteroids to the management of AAV had dramatically changed the outcome of AAV, although cure remains uncommon.^14^ Recently, rituximab (anti CD20 monoclonal antibody) has emerged as a new treatment option for the induction as well as for maintaining remission of AAV.^15^

The 5-year survival of treated AAV is over 70%, but relapse rate remains relatively high.^14–17^ A systematic review by the European League Against Rheumatism systemic vasculitis task force^16^ has revealed that the 5-year survival for GPA, MPA, and EGPA was 74% to 91%, 45% to 76%, and 60% to 97%, respectively. The rate of renal survival in GPA varied from 23% at 15 months to 23% at 120 months.^16^ Factors influencing remission, relapse, renal, and patient survival include immunosuppressive drugs, type of organ involvement, presence of ANCA, older age, and male gender.^17^ An increase in age of 10 years was significantly associated with poor renal survival (hazard ratio [HR] 1.47 (95% CI 0.95–2.24, *P* = 0.08) as well as overall patient survival (HR 3.4, 95% CI 1.03–11.21, *P* = 0.04).^17^ A recent study has demonstrated a bimodal pattern of mortality in GPA: an early mortality that is primarily attributed to infection, active vasculitis, and renal failure, and a late mortality that is speculated to be associated with the burden of cardiovascular disease and the toxic effects of the immunosuppressive drugs used to treat patients with AAV.^18^ Advanced age at the time of diagnosis of AAV has a detrimental influence on the outcome and survival of patients with AAV.^19^ The predictive values of variables associated with mortality were analyzed in a Chinese cohort of 398 consecutive patients with AAV.^20^ During follow-up of a median duration 25.5 months, 33.9% of the patients have died. In accordance with our results, independent predictors of all-cause mortality at baseline included age (increased by 10 years; HR 1.8, 95% CI 1.512–2.142, *P* < 0.001) and initial renal function (increase of 24-hour creatinine clearance rate by 10 mL/min; HR 0.889, 95% CI 0.832–0.950, *P* = 0.001).

The peak age of patients with AAV is 65 to 74 years,^21^ and it is expected that patients at such age will suffer of other diseases, including hypertension, diabetes mellitus, and cardiovascular, at the time they are diagnosed with AAV.^22^

The CCI,^23^ a method of predicting mortality by classifying or weighting comorbid conditions (comorbidities), has been widely utilized by health researchers to measure burden of disease. The reliability of the CCI comes from its ability to predict major morbidity and mortality.^24–26^ The method was developed from a cohort of 604 hospital patients in the United States in order to predict 1-year mortality in patients with cancer and was validated in a cohort of 685 women with breast cancer followed for 10 years.^23^ Moreover, the CCI had been validated for its ability to predict mortality in various diseases, including cancer, renal disease, stroke, patients admitted to an intensive care, and liver disease.^27–30^ The method proposed by Charlson was adapted to obtain data on comorbidities, coded according to the International Classification of Diseases, 9th revision.^31^

We, therefore, aimed to evaluate the influence of comorbidity at the time of AAV diagnosis on renal outcome and patient survival in our cohort of patients who were diagnosed and followed at a single center. For the purpose of the study, we have used the CCI adjusted for collagen vascular disorders and renal failure as well as for age. We found that higher CCI (a) at diagnosis was significantly associated with a higher disease activity score (BVAS) (*P* = 0.02) as well as with increased risk for accrual of CKD (eGFR ≤60 mL/min/2.73 m^2^; *P* = 0.036) and mortality (*P* = 0.014).

**FIGURE 1:**
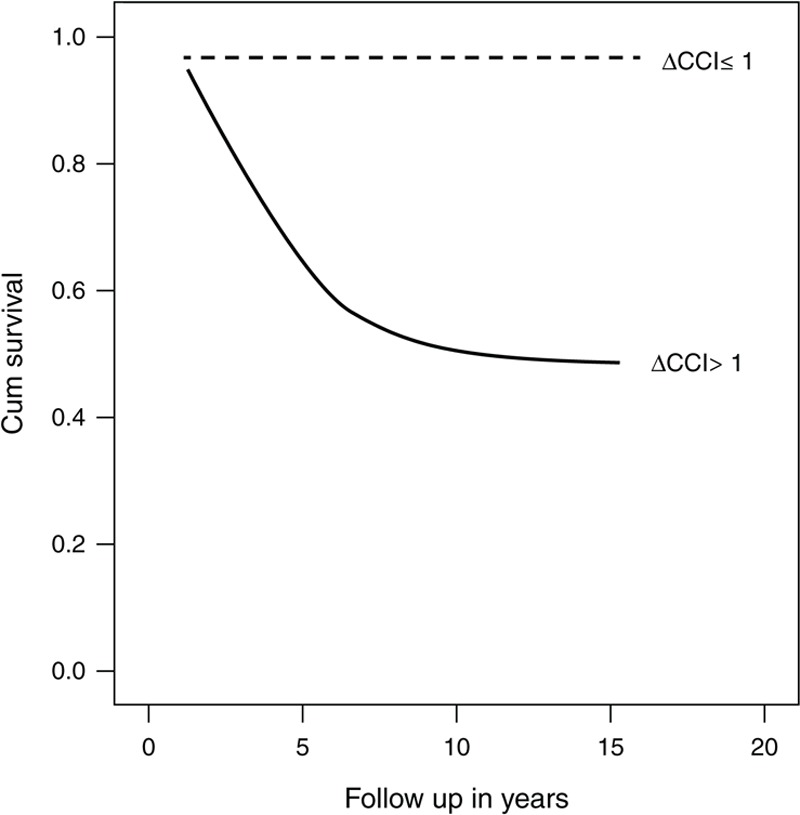
Kaplan–Meier curve showing an association between ΔCCI and all-cause mortality in a cohort of patients with ANCA-associated vasculitis (*P* = 0.002). ANCA = antineutrophil cytoplasmatic antibody, ΔCCI = the difference of CCI between the last and first visits.

**FIGURE 2:**
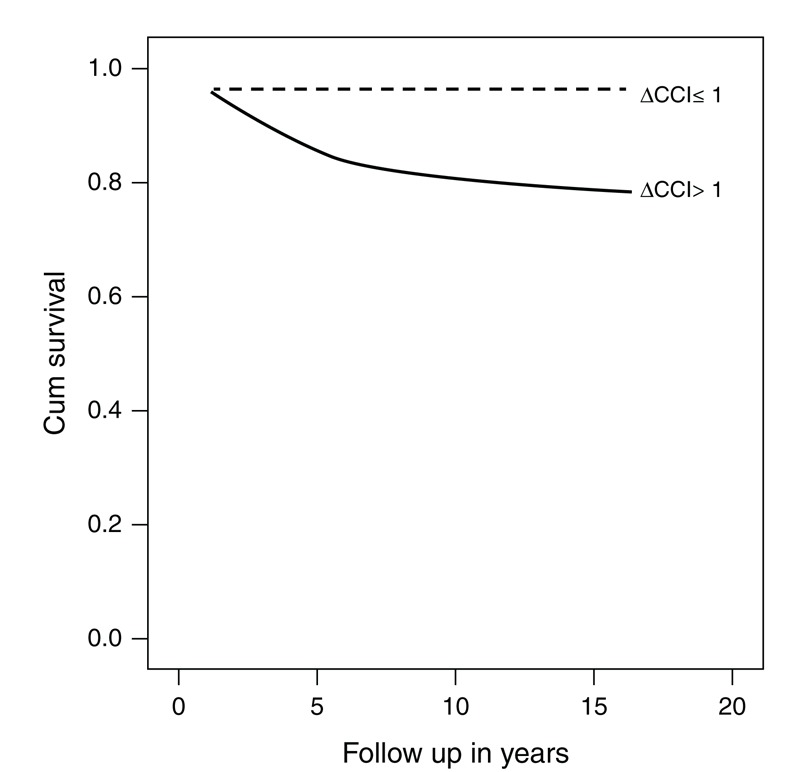
Kaplan–Meier curve showing the association between ΔCCI and estimated GFR (>60, first line; <60, second line) deterioration (*P* = 0.036). ΔCCI = the difference of CCI between the last and first visits.

An increment of ≥1 of CCI (a) at the last encounter is correlated with reduced renal and patient survival (Figures 1 and 2). Our results are in line with the results found in patients with rheumatoid arthritis^6,7^ and hemodialysis.^32–35^

Our study has several limitations. The CCI was retrospectively calculated, although the fact that all the patients who were recruited into our cohort were diagnosed, treated and continuously followed at our hospital and the use of computerized database of the hospital may have mitigated some of this effect.

Our results strengthen the grave prognostic impact of comorbidities on the outcome of AAV and suggest that CCI should be scored at the time of diagnosis as well at disease relapse and routinely included in the assessment of patients with AAV.
